# Supplementary material for: Structure of the reduced microsporidian proteasome bound by PI31-like peptides in dormant spores
Source: Nat Commun. 2022 Nov 15;13:6962. doi: 10.1038/s41467-022-34691-x (PMC9666519; doi:10.1038/s41467-022-34691-x)
Supplement: Supplementary file 1 — Supplementary Information [file 41467_2022_34691_MOESM1_ESM.pdf]

Supplementary Information

# Structure of the reduced microsporidian proteasome bound by PI31-like peptides in dormant spores.

Nathan Jespersen[1,3], Kai Ehrenbolger[1,3], Rahel R. Winiger[1,3], Dennis Svedberg[1], Charles R. Vossbrinck[2], Jonas Barandun[1,*]

[1] Department of Molecular Biology, The Laboratory for Molecular Infection Medicine Sweden (MIMS), Umeå Centre for Microbial Research (UCMR), Science for Life Laboratory, Umeå University, 90187 Umeå, Sweden

[2] Department of Environmental Science, Connecticut Agricultural Experiment Station, New Haven, Connecticut 06504, USA

[3] These authors contributed equally to this work.

[*] Corresponding author: jonas.barandun@umu.se. (J.B.)

**Supplementary Table 1.** Cryo-EM data collection, refinement, and model statistics.
**Supplementary Table 2.** *V. necatrix* proteasome subunit sequences and model composition.

**Supplementary Figure 1.** Cryo-EM data processing scheme.
**Supplementary Figure 2**. Overall and local resolution estimation.
**Supplementary Figure 3.** Identification and model of the microsporidian PI31-like peptide.
**Supplementary Figure 4**. Conformational variation of the Met45 residue in b5 subunits.

**Supplementary Table 1. Cryo-EM data collection, refinement, and model statistics.**

| | **20S Spores** | **20S Sporoplasm** | **26S Sporoplasm** |
|---|---|---|---|
| | EMD-15365<br>PDB- 8ADN | EMD-15367 | EMD-15366 |
| **Data collection and processing** | | | |
| Voltage (kV) | 300 | 300 | 200 |
| Pixel Size (Å) | 1.042 | 1.042 | 1.495 |
| Electron exposure (e-/Å2) | 35.01 | 42.31 | 40.0 |
| Defocus range (um) | 0.7 – 2.8 µm | 1.2 - 3.0 µm | 1.2 - 3.0 µm |
| Symmetry imposed | C2 | C2 | C1 |
| Final particle images | 52,679 | 17,942 | 6,442 |
| Resolution (Å) | 2.8 | 3.2 | 8.3 |
| FSC threshold | 0.143 | 0.143 | 0.143 |
| Map sharpening B-Factor (Å2) | 68.3 | 66.1 | 514.1 |
| | | | |
| **Refinement** | | | |
| Initial model used | 5CZ4 | | |
| Model composition | | | |
| Non hydrogen Atoms | 48,318 | | |
| Protein residues | 6,160 | | |
| R.m.s deviations | | | |
| Bond length (Å) | 0.004 | | |
| Angles (°) | 0.927 | | |
| Validation | | | |
| MolProbity score | 1.32 | | |
| Clashscore | 4.93 | | |
| Poor rotamers (%) | 0.85 | | |
| Ramachandran | | | |
| Favored (%) | 97.73 | | |
| Allowed (%) | 2.27 | | |
| Outliers (%) | 0.00 | | |
| Ramachandran plot Z-scores | | | |
| whole | 0.68 | | |
| helix | 1.43 | | |
| sheet | 0.39 | | |
| loop | -0.29 | | |

**Supplementary Table 2. *V. necatrix* proteasome subunit sequences and model composition.**

| **Subunit** | **Chains *SeqIDs*** | **Sequence** (Propetide, **Active site threonine**, Sequence not observed, Modelled, Sidechains trimmed) |
|---|---|---|
| PSA1 | G,U | MSIKDEIYNIFNADGKILQIEYGLEAVNKSLPLVVLKNKNMIVCAAKKNQGHLLEDEVQTSFQPIYPNLYSAFTGNWADVFYVNSKAKDLAHYASYKLGFSVTPDILCRKLADELQPLIQSTGERAPAFAGALFGFDNGKPVVYMTNISAVCYPVYGSMVGSKNQNMYKYVEKYYNEDIEDEKLFEVAVGGLLESLGENSVYQEMEVAYLRNGEVLKYLDDKEIESLLLSIADK |
| PSA2 | A,O | MFLDTHKQLTLFTSEGKLDQCDNALKAATQGSLSVGCVSENGVVLASLKESNNLVILSEYKKIYQISPNLGITYSGCQPDFRIQYNLSLKISEEYTDIYSTNIPIRLFVEQFSRQIQEYTIKKGYRPFGTLLLIVGDNKMYRVDPSGSYTSLQVGTIGREYTESGRLLERRKGMLDDNISTCVECIREYCGRSVKSEDIDIGVYRGQEFRVYSKEEVQEVFDSINKI |
| PSA3 | B,P | MSSNTFTEEGRLLQTEYAIKNVSKGGTIIGLVCKDGVILLGINKTELLDEREKIYKINPKVYVSVSGLFGDAMLLKKYGQVKAQDFLYEFDYDCDIDRICNFISEKKQLFTQYNSTRPFGFSFIYAGMKNNKFKLCSTDPSGTINEWKGVCFGENEDAINNGLRNDFPDEEMDMERGLFEIFKILSKVTECSAKDHKKYEILYFKNEESRFLEFEEIENILLRIEEEKNKK |
| PSA4 | C,Q | MEYENALGIFSPDGRLIQVEYAQQASEQGSLVVFSSDTNEICLSIETKTHNKMLIDQNKLLPVDKDLNIWYTFSGIKPDSYKVLNEARLICRNYKIKTGTNISFDELAYELSLYKQKFTLDSSMRPFGIRSILLQVKDMAKIYVLEPDGNYSEYKCGAVGQKSVSVCEYLEKCEEEDIIFRSVSGLGTVVQSDKNKVMSYVISKDEIRRVEDETVSQIISTVSVK |
| PSA5 | D,R | MSIVSRQNANTYSAEGRLYQVEYAMQAMNLGTSSIGIKTKDYVLLASEKKIISKLQNPSSVKKHYRVYDHIALGFSGISADVKTIVDKSRNFAINHEYLYDENCKVERLLEHLADLSLNFDKKEADEKIFSRPFGASLLIIGYDTEPRLFSLDPSGSYLEYHAKAIGSGSEVIENMLEQEFDPNVDINSGLKNILNMLSKVMKDKINNFNVEITAITKNECKILTPEEIEQFLE |
| PSA6 | E,S | MNSQTDYTNYIIFNPEGKIKQLEFINNTVQLGSTVVALKNKSFGVFVTYNEKRSKFALQQKKIFPINSKSLFSFSGITNDGTKIVKYLKNSTVFENIRKGRDIHPIHVFDDLCYSACIRTLTNGNRLYGVQGLLLTDYQGISLVLFDPKGSAKEVRGMSIGSRSQSCRTILEDECDKFEEYNKEELVRLGIKALRNAYPETGVLNKDNVDIWILETNQESKQIKSEEYLQ |
| PSA7 | F,T | MAILDVDTVYTNTGDILQIGYAQKAADNGNTAICMKNKKGLIMIAEKPIESKLYVSEKNFRIKKVNNSIFQISSGIETDLVYINENLKNNLISEKHSNDMDVSHESVRNQIVNIIHQFTRYSGVRPIGINLLTCSKYKNEYKILQTDCTGKSLFFKSSVIGKGSRIVKTELEKLNLENMEIRDLVENGIRILYKSYDPLKDKPFDIEIGIMCEETNGEFVRLEKNQYSEIIEKYKDFSVDGEE |
| **PSB1** | N,b | MVAYDNKNNEGFSPNLPEMTG**T**TIMAVKYADGILIGADCRTSMGTYVSSRFTDKLTKISDNIYCCRSGSAADTQAITQYITELVQRSSFIDKEIPSVKKAAMAAKDIIYRYPNMLAGLIIAGYDTKPRIFNISLGGTMTEAEWQIGGSGSAYIYGLCDTTFKPNMNLEEALEFVKLAVTCAIKRDNASGGCIRMASITREGVQRFFYSGDKILNST |
| **PSB2** | H,V | MFTKTG**T**TIVGVKYKGGVVICADTRSTSGPIVADKNCSKIHYISDNIQACGAGTSADITRVTRKASKVLSIFSKTYNRLPRVSHCVRTCQLHLHPYQGHISAALVVGGVDDTGAHLYDVYPHGSSNSVSYTALGSGSLAAISILEAGYKDMNREEAMELACAAIEAGIMNDLYSGSNIDVCVISQEGREMFRNYKKVGVRNEVPKFQYPRSSVKILKEDIYKYIEEK |
| PSB3 | I,W | MSDISQHYGGSLLAMIGKSSVAFLSDKRLGSGPISVSKNFTKIYSLTPRLFFGFTGLVSDGEMLFKKIRKNYNLFVQDNNKDMEPSELSNMISYILYQKRLQPYYVAVIVCGMTLDKKPYASSMDCIGAMKETSEFVTSGTASKNLMGLSEALFYPEMEDEDLFTTSVQTFLNSSDRDTFGGMGFECLLINPEGYKRREFVGRCD |
| PSB4 | J,X | MESSVALKGNDFVIIGTDSSVKNSYLVLKREEDKFYNINNKVVFTYLGDQGDAFRTSSFINEKLVYEEIQNNVEITPKVTANVIQKTLYDNLRSHPKNCYFLVGGLSQDGPELYSVDLYGSLHENDFMAVGISTYFCYGVLDKEYHKNITKEDGIKIIQKCFDVLKQRCSVDISNIEIKIVSKEGVETINKVL |
| **PSB5** | K,Y | MEKLFTGDIMEIQNTKVDSAFMKNKIVPYKG**T**TTLAFIFQGGMVIAVDSRASAGSYIASQNVHKVIRVNKHLIGTMAGGASDCYFWEKKMGLYAKLYELKNNKRISVSAASMYLSNCVYSYKGQGLSLGSMVCGYDGDKPVIYYVDDAGQRLSGDLFSVGSGSTIAYGVLNESYRFDLTKEEALNLGKKAIWHATHRDAYSGGNVNLYFMDKNGWEHLGTFDVDKFEQ |
| PSB6 | L,Z | MFTLQANTKDQIIKDLNIGDLTIRDLNIKDLNIGDSSTVNLFKVDIPLLSNEIPLDFTFDNLPSNKKEIFESFDSFTDFVEGKTKSQESKFNPYEDNSGSTVSIRLNNSIIIAADTRHCSEMGIYSRNTSKIFRIGDFLLTITGFYADGYELYNRLKYQVQIYESFNKISIHSLANLASKIMYSKRLFPYYSYVTLSGFEGDNPYVYSFDCLGHFEEVDSVCNGSGSPLIQPLLDSTIEKKNWAGENYEVTEEYVKDIVRRGFNAASERDVKTGDNVEIWIIKKDGMTKEYERLRQD |
| PSB7 | M,a | MKNFVIGSGVISLRYKNGVITCTDTQASYGNLCKFNDVRRIFRLSSNTLISLSGEISDIQFLMNELNKLNESDPVKMSPRGYLNLVQGILYNKRSRVEPLNVSVSIVGVDDDDFLVSCVNHLGNFYEDNIVCTGLSNMIALPFLRTCNVLDLERDEAISLVEKAMTVMCYRSCRSSNRIQIGVVEKGLVDISDPYVLNTDWQVGHNEEEIVL |
| PI31L | 3,4 | MDFQDYIQSLKKDFKLVKINDHTYILHKNKKTLELTPDKMYNIQEINDILNVSYPDISYDRNLEDLGSKNKGILNGFGNVGEDDLHPQIGRRKGKKKGAIFSPEEFKEEEDSDGIDKTDIFPLKKKRDPDSDHFKKTGGDDDNPFLY |

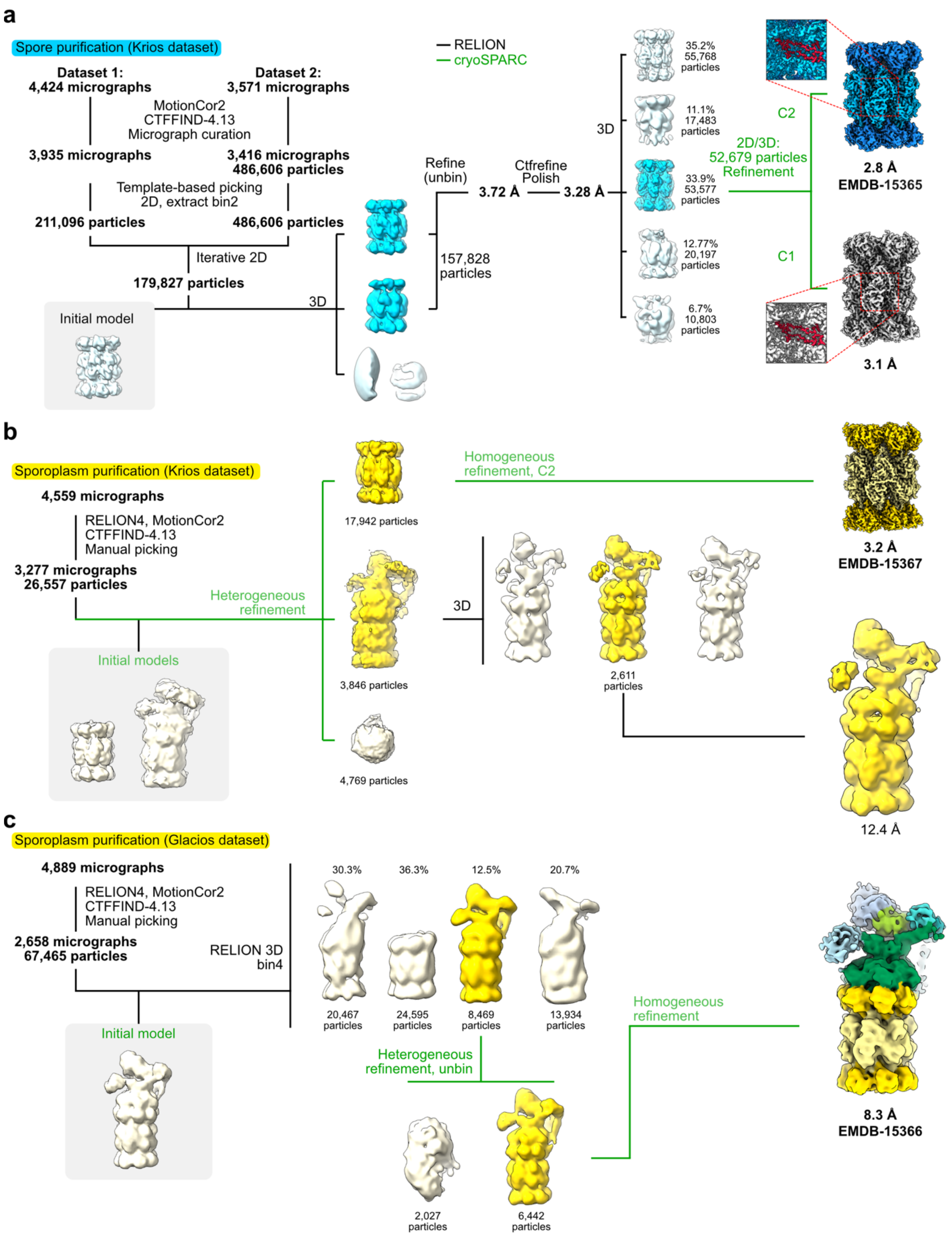


**Supplementary Figure 1. Cryo-EM data processing scheme**. **a-c** Data collection and processing procedures for the two spore-derived datasets, the sporoplasm-derived 20S dataset, and the sporoplasm-derived 26S dataset, respectively. Steps performed in cryoSPARC[1] are represented by green typeface, while steps in Relion[2] are denoted by black typeface.

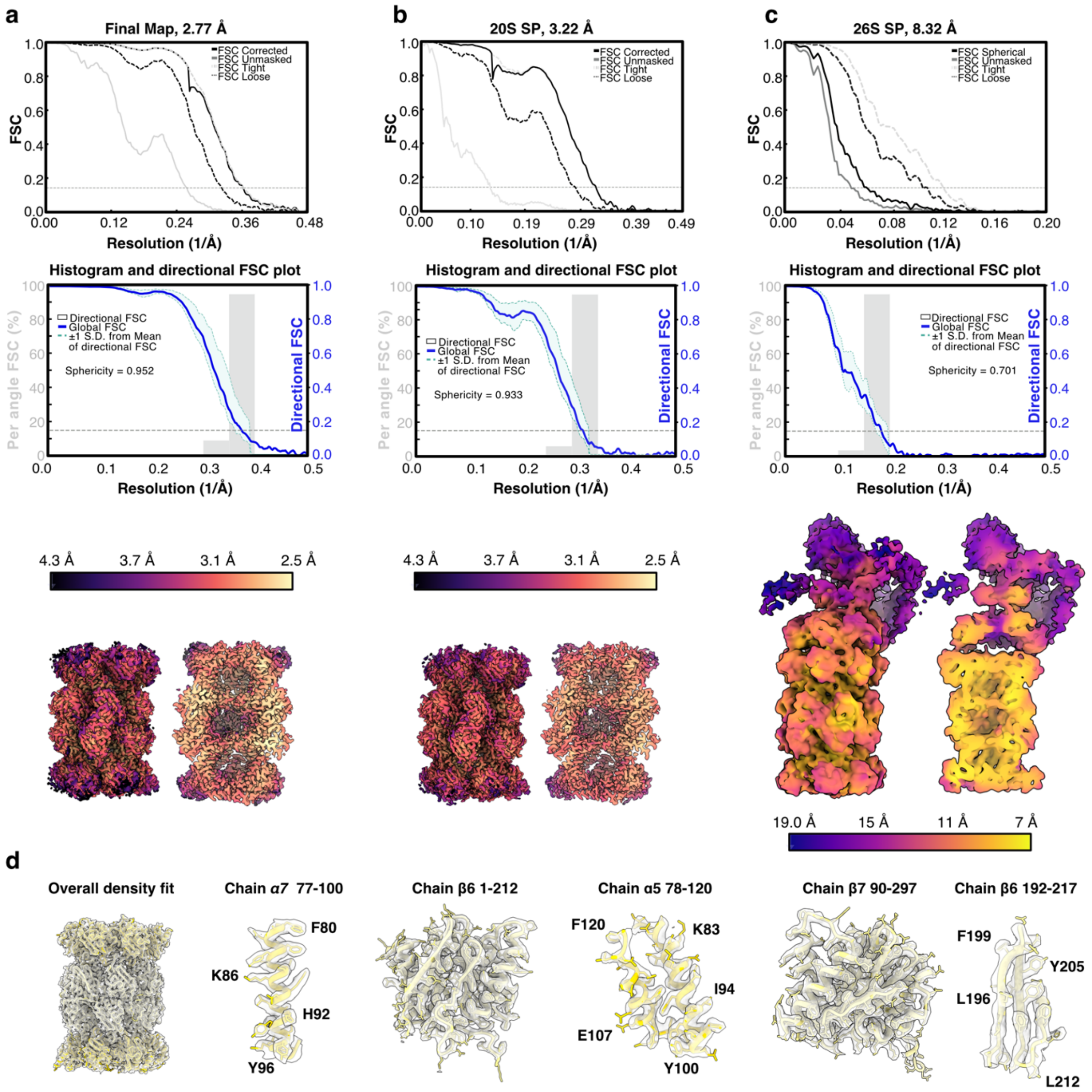


**Supplementary Figure 2**. **Overall and local resolution estimation. a-c** Overall and local resolution estimation of the spore derived 20S (a), the sporoplasm derived 20S (b) and the sporoplasm derived 26S proteasome. (a-c), top to bottom: The cryoSPARC generated Fourier Shell Correlation (FSC) curves are shown above the corresponding 3DFSC plot. The bottom row displays the local resolution surface representations of the respective maps (left) and their corresponding slap view (right) **d** Density and model fit examples of the full spore 20S map, single chains, and selected areas. Residue range and single residues are indicated.

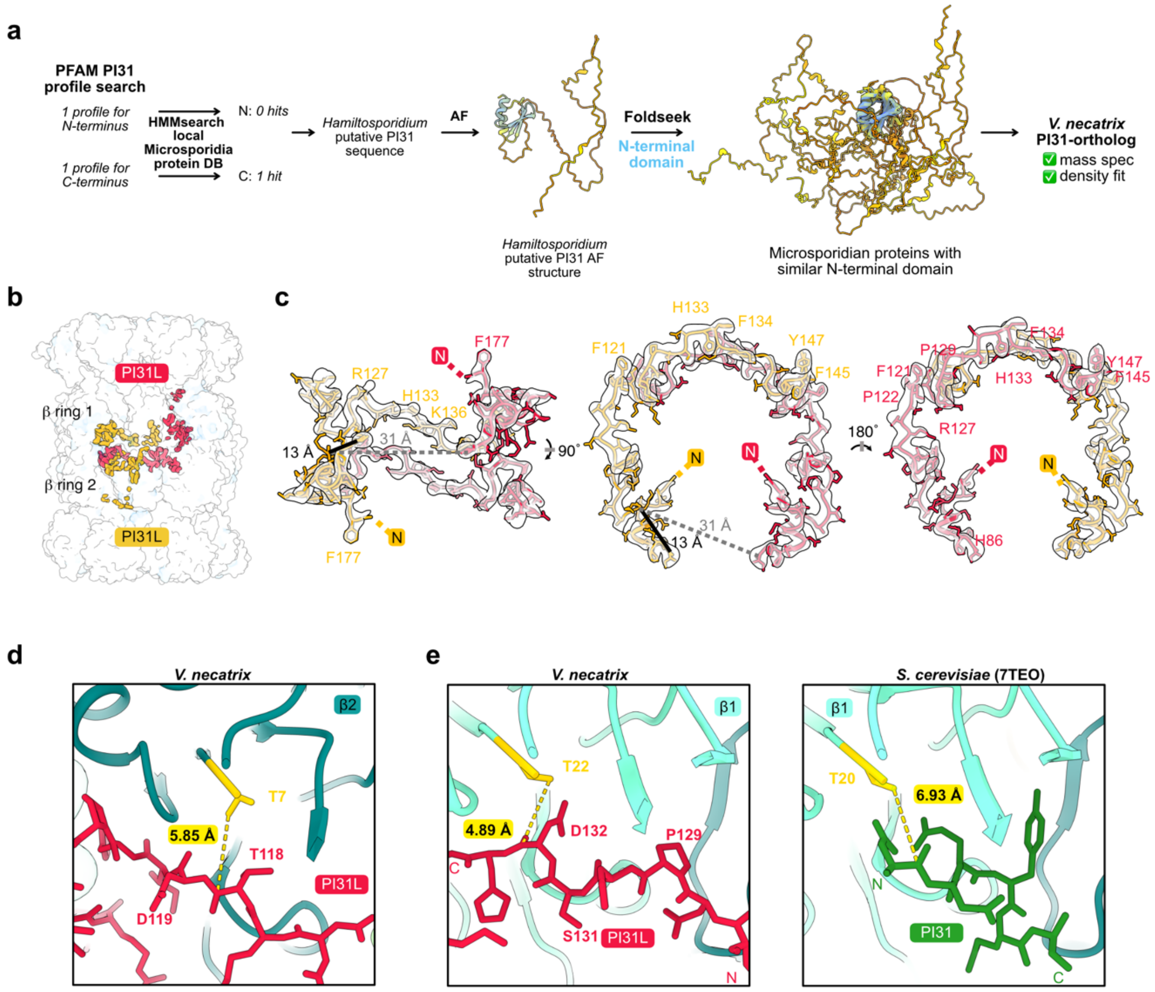


**Supplementary Figure 3. Identification and model of the microsporidian PI31-like peptide. a** A PI31 Pfam search for HMM profiles led to the identification of an N-terminal and C-terminal domain. These HMM profiles were used for an HMM search against a local microsporidian database consisting of all microsporidian sequences available on UniProt. The C-terminal profile resulted in a hit from *H. magnivora* (UniProt A0A4Q9M0M2). The protein was folded using AlphaFold and used as a query for Foldseek, capitalizing on the DeepMinds new UniProtKB database that includes all microsporidian predictions combined with a local *V. necatrix* structure database. This structural search successfully identified the *V. necatrix* PI31-like protein, which was further validated by mass spectrometry data and a high-quality cryo-EM density fit. **b** Overview of the two PI31-like peptides (orange, red) in the active sites of the 20S proteasome (outlines). **c** Density/model fit of the PI31-like peptides, with selected residues labeled. A black solid and dashed grey line displays the distance between residue 89 and 96 within PI31 or between protomers, respectively. **d, e** Active site details of PI31L (chain 4) bound to β2 (d) and β1 (e) in *V. necatrix* next to the same view in *S. cerevisiae* (PDB 7TEO[3]).

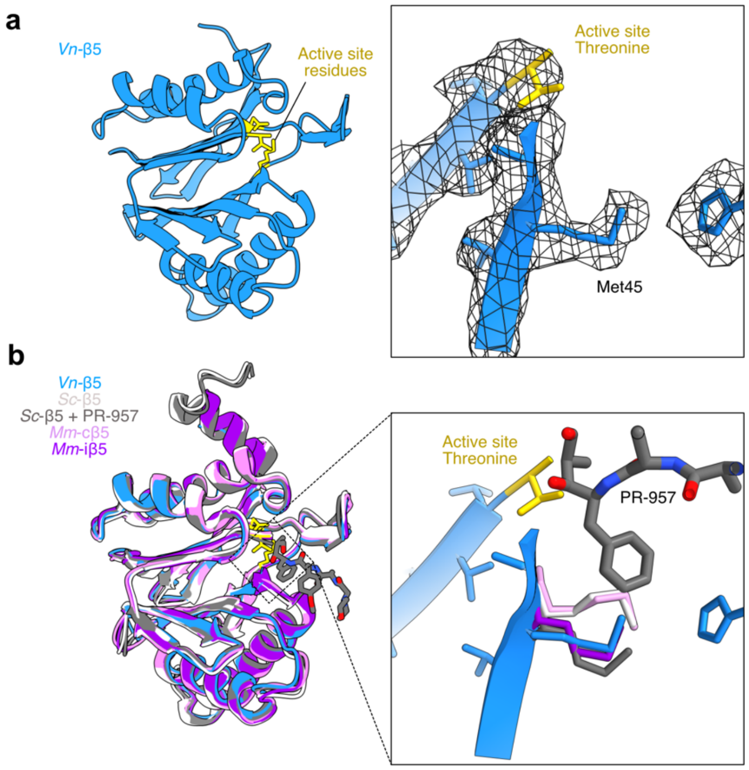


**Supplementary Figure 4**. **Conformational variation of the Met45 residue in β5 subunits. a** Cryo-EM model and electron density mesh map (right) for the *V. necatrix* β5 subunit. Active site residues Thr1, D17, and Lys33 are highlighted in gold, with sidechains shown as sticks. Residues surrounding Met45 are removed for clarity. **b** Superposition of β5 subunits from *V. necatrix* (blue), *S. cerevisiae* (PDB 5CZ4[4], light grey), *S. cerevisiae* in complex with the PR-957 inhibitor (3UN4[5], dark grey), the *Mus musculus* constitutive proteasome (PDB 3UNE[5], pink), and the *M. musculus* immunoproteasome (PDB 3UNH[5], purple). A rotated and magnified superposition of the Met45 residues (right) demonstrates the distinct conformational change to Met45 seen in *S. cerevisiae* upon PR-957 binding. A similar conformation difference is associated with selective binding of the inhibitor to *M. musculus* immunoproteasomes over constitutive proteasomes.

**Supplementary References**


1. Punjani, A., Rubinstein, J. L., Fleet, D. J. & Brubaker, M. A. CryoSPARC: Algorithms for rapid unsupervised cryo-EM structure determination. *Nat Methods* **14**, 290–296 (2017).
2. Kimanius, D., Dong, L., Sharov, G., Nakane, T. & Scheres, S. H. W. New tools for automated cryo-EM single-particle analysis in RELION-4.0. *Biochem J* **478**, 4169–4185 (2021).
3. Rawson, S. *et al.* Yeast PI31 inhibits the proteasome by a direct multisite mechanism. *Nat Struct Mol Biol* **29**, 791–800 (2022).
4. Huber, E. M. *et al.* A unified mechanism for proteolysis and autocatalytic activation in the 20S proteasome. *Nat Commun* **7**, 10900 (2016).
5. Huber, E. M. *et al.* Immuno- and Constitutive Proteasome Crystal Structures Reveal Differences in Substrate and Inhibitor Specificity. *Cell* **148**, 727–738 (2012).
