# Supplementary material for: Structure of the reduced microsporidian proteasome bound by PI31-like peptides in dormant spores
Source: Nat Commun. 2022 Nov 15;13:6962. doi: 10.1038/s41467-022-34691-x (PMC9666519; doi:10.1038/s41467-022-34691-x)
Supplement: Supplementary file 3 — Description of Additional Supplementary Files [file 41467_2022_34691_MOESM3_ESM.pdf]

File name: Supplementary Data 1

Description: Mass spectrometry data for proteasome samples derived from dormant spores. As the available *Vairimorpha necatrix* genome is not yet annotated, potential identities for hits were defined via a pblast against yeast proteomes. Proteins with no pblast hit above a cut-off E-value of 0.05 were considered not-identifiable (*N.I.*). Proteasome CP subunits are highlighted in green, and incorrectly identified yeast hits for CP proteins are noted in red. The PI31-like protein bound to spore 20S proteasomes is highlighted in purple.

File name: Supplementary Data 2

Description: Protein sequences for proteasome subunits in microsporidians with available genome assemblies and for selected outgroups. Sequences for *V. necatrix* are derived from an in-house genome assembly (manuscript in preparation). Yeast and *V. necatrix* sequences are aligned to demonstrate the frequent deletion of protein segments in microsporidia.
