# Supplementary material for: Structure of the reduced microsporidian proteasome bound by PI31-like peptides in dormant spores
Source: Nat Commun. 2022 Nov 15;13:6962. doi: 10.1038/s41467-022-34691-x (PMC9666519; doi:10.1038/s41467-022-34691-x)
Supplement: Supplementary file 6 — Reporting Summary [file 41467_2022_34691_MOESM6_ESM.pdf]

nature portfolio

Corresponding author(s): Jonas Barandun

Last updated by author(s): Nov 1, 2022

Reporting Summary

Nature Portfolio wishes to improve the reproducibility of the work that we publish. This form provides structure for consistency and transparency in reporting. For further information on Nature Portfolio policies, see our Editorial Policies and the Editorial Policy Checklist.

Statistics

For all statistical analyses, confirm that the following items are present in the figure legend, table legend, main text, or Methods section.

n/a	Confirmed	
☐	☒	The exact sample size (*n*) for each experimental group/condition, given as a discrete number and unit of measurement
☐	☒	A statement on whether measurements were taken from distinct samples or whether the same sample was measured repeatedly
☒	☐	The statistical test(s) used AND whether they are one- or two-sided<br>*Only common tests should be described solely by name; describe more complex techniques in the Methods section.*
☒	☐	A description of all covariates tested
☒	☐	A description of any assumptions or corrections, such as tests of normality and adjustment for multiple comparisons
☐	☒	A full description of the statistical parameters including central tendency (e.g. means) or other basic estimates (e.g. regression coefficient) AND variation (e.g. standard deviation) or associated estimates of uncertainty (e.g. confidence intervals)
☒	☐	For null hypothesis testing, the test statistic (e.g. *F*, *t*, *r*) with confidence intervals, effect sizes, degrees of freedom and *P* value noted<br>*Give P values as exact values whenever suitable.*
☒	☐	For Bayesian analysis, information on the choice of priors and Markov chain Monte Carlo settings
☒	☐	For hierarchical and complex designs, identification of the appropriate level for tests and full reporting of outcomes
☒	☐	Estimates of effect sizes (e.g. Cohen's *d*, Pearson's *r*), indicating how they were calculated

Our web collection on statistics for biologists contains articles on many of the points above.

Software and code

Policy information about availability of computer code

Data collection	EPU v2.8.0, EPU v2.13
Data analysis	Relion (v3.1), Relion (v4.0), MotionCor2, CTFFIND (v4.1.14), cryoSPARC (v3.3.2), Coot 0.8.9.3, PHENIX (v1.20-4487), ChimeraX (v1.3), ISOLDE (v1.3), Foldseek (v3-915ef7d), ColabFold (v1.3.0), MUSCLE5 (v5.1), trimAl (v1.4.1), FASconCAT-G (v1.05.1), iqtree (v2.2.0), Figtree (v1.4.4)

For manuscripts utilizing custom algorithms or software that are central to the research but not yet described in published literature, software must be made available to editors and reviewers. We strongly encourage code deposition in a community repository (e.g. GitHub). See the Nature Portfolio guidelines for submitting code & software for further information.

Data

Policy information about availability of data

All manuscripts must include a data availability statement. This statement should provide the following information, where applicable:

- Accession codes, unique identifiers, or web links for publicly available datasets
- A description of any restrictions on data availability
- For clinical datasets or third party data, please ensure that the statement adheres to our policy

The cryo-EM density maps, the coordinates, and the raw micrographs for the microsporidian proteasome have been deposited in the EM Data Bank (https://www.ebi.ac.uk/pdbe/emdb/), the Protein Data Bank (https://www.rcsb.org), and the Electron Microscopy Public Image Archive (https://www.ebi.ac.uk/pdbe/emdb/

empiar/) with accession codes EMD-15365 [https://www.ebi.ac.uk/emdb/EMD-15365], PDB-8ADN [https://www.rcsb.org/structure/8ADN], and EMPIAR-11117 [https://www.ebi.ac.uk/empiar/EMPIAR-11117/] for the spore stage 20S core-particle, EMD-15367 [https://www.ebi.ac.uk/emdb/EMD-15367] and EMPIAR-11118 [https://www.ebi.ac.uk/empiar/EMPIAR-11118/] for the sporoplasm-derived 20S core-particle (post-germination), and EMD-15366 [https://www.ebi.ac.uk/emdb/EMD-15366] and EMPIAR-11121 [https://www.ebi.ac.uk/empiar/EMPIAR-11121/] for the full 26S proteasome. The yeast proteasome structures (PDB-5CZ4 [https://www.rcsb.org/structure/5CZ4]) and (PDB 6FVU [https://www.rcsb.org/structure/6FVU]) were used as templates.

Human research participants

Policy information about studies involving human research participants and Sex and Gender in Research.

Reporting on sex and gender	N/A
Population characteristics	N/A
Recruitment	N/A
Ethics oversight	N/A

Note that full information on the approval of the study protocol must also be provided in the manuscript.

Field-specific reporting

Please select the one below that is the best fit for your research. If you are not sure, read the appropriate sections before making your selection.

☒ Life sciences ☐ Behavioural & social sciences ☐ Ecological, evolutionary & environmental sciences

For a reference copy of the document with all sections, see nature.com/documents/nr-reporting-summary-flat.pdf

Life sciences study design

All studies must disclose on these points even when the disclosure is negative.

Sample size	The sample size of the cryo-electron microscopy datasets were determined by allocation of measurement time on microscope and the particle density on the cryo-EM grid. For multiple sequence alignments and phylogenetic tree calculations, one gene per subunit per organism was selected.
Data exclusions	The micrographs have been evaluated for quality and micrographs with drift, poor CTF fits and ice of low quality have been removed according to established standard procedures.
Replication	No replication studies were attempted as the solved structure compares well with previous solved cryo-EM structures of proteasomes. The cryo-EM map was calculated according to standard procedures.
Randomization	For 3D refinement, the dataset was split into two independent randomized halfsets. Randomization is not relevant for multiple sequence alignments or phylogenetic tree calculations as there was only one type of sample/dataset.
Blinding	To avoid model bias, we used a cryoSPARC de-novo generated low-pass filtered initial model of the microsporidian proteasome, and performed standard procedures to ensure maps/models were not overfit, as described in the Methods section. Blinding is not relevant for cryoEM refinement, sample characterization, multiple sequence alignments or phylogenetic tree calculations as there was only one type of sample/dataset.

Reporting for specific materials, systems and methods

We require information from authors about some types of materials, experimental systems and methods used in many studies. Here, indicate whether each material, system or method listed is relevant to your study. If you are not sure if a list item applies to your research, read the appropriate section before selecting a response.

Materials & experimental systems

n/a	Involved in the study
☒	☐ Antibodies
☒	☐ Eukaryotic cell lines
☒	☐ Palaeontology and archaeology
☒	☐ Animals and other organisms
☒	☐ Clinical data
☒	☐ Dual use research of concern

Methods

n/a	Involved in the study
☒	☐ ChIP-seq
☒	☐ Flow cytometry
☒	☐ MRI-based neuroimaging
